# Arrhythmogenic Potential of Myocardial Edema: The Interstitial Osmolality Induces Spiral Waves and Multiple Excitation Wavelets

**DOI:** 10.3390/biomedicines12081770

**Published:** 2024-08-06

**Authors:** Diana G. Kiseleva, Vitalii D. Dzhabrailov, Aleria A. Aitova, Elena A. Turchaninova, Valeriya A. Tsvelaya, Maria A. Kazakova, Tatiana Yu. Plyusnina, Alexander M. Markin

**Affiliations:** 1 Laboratory of Cellular and Molecular Pathology of Cardiovascular System, Petrovsky National Research Centre of Surgery, 119991 Moscow, Russia; alexander.markin.34@gmail.com; 2 Department of Biophysics, Faculty of Biology, Lomonosov Moscow State University, 119991 Moscow, Russia; plusn@yandex.ru; 3 ITMO University, 191002 Saint-Petersburg, Russia; vitalyadd22000022@gmail.com (V.D.D.); vts93@yandex.ru (V.A.T.); 4 Moscow Center for Advanced Studies, Kulakova Str. 20, 123592 Moscow, Russia; 5 M.F. Vladimirsky Moscow Regional Clinical Research Institute, 129110 Moscow, Russia; 6 Department of Biophysics, Faculty of Physics, Lomonosov Moscow State University, 119991 Moscow, Russia; maria-kazakova99@yandex.ru; 7 Medical Institute, Peoples’ Friendship University of Russia named after Patrice Lumumba (RUDN University), 117198 Moscow, Russia

**Keywords:** excitation wavelets, spiral waves, re-entry, ventricular fibrillation, ventricular arrhythmia, edema, osmolality, cell swelling, myocardial infarction, optical mapping

## Abstract

Myocardial edema is a common symptom of pathological processes in the heart, causing aggravation of cardiovascular diseases and leading to irreversible myocardial remodeling. Patient-based studies show that myocardial edema is associated with arrhythmias. Currently, there are no studies that have examined how edema may influence changes in calcium dynamics in the functional syncytium. We performed optical mapping of calcium dynamics on a monolayer of neonatal rat cardiomyocytes with Fluo-4. The osmolality of the solutions was adjusted using the NaCl content. The initial Tyrode solution contained 140 mM NaCl (1T) and the hypoosmotic solutions contained 105 (0.75T) and 70 mM NaCl (0.5T). This study demonstrated a sharp decrease in the calcium wave propagation speed with a decrease in the solution osmolality. The successive decrease in osmolality also showed a transition from a normal wavefront to spiral wave and multiple wavelets of excitation with wave break. Our study demonstrated that, in a cellular model, hypoosmolality and, as a consequence, myocardial edema, could potentially lead to fatal ventricular arrhythmias, which to our knowledge has not been studied before. At 0.75T spiral waves appeared, whereas multiple wavelets of excitation occurred in 0.5T, which had not been recorded previously in a two-dimensional monolayer under conditions of cell edema without changes in the pacing protocol.

## 1. Introduction

Myocardial edema, defined as the accumulation of fluid in interstitial and/or intracellular compartments, is observed in coronary heart disease, myocardial infarction, myocarditis, and various types of cardiomyopathies, covering most of the widespread cardiovascular diseases among the population [1]. 

Currently, there is no drug therapy that might effectively address myocardial edema, due to the differences in its location and volume. In addition, changes in the osmolality of interstitial and intracellular fluids have different etiologies, which also complicates the development of a unified approach for treatment [2,3,4]. As for systemic edema, which occurs, for example, in heart failure, it can be effectively eliminated with diuretics that remove excess fluid from the body through the urinary tract [5]. However, such drugs are ineffective for local myocardial edema and do not eliminate its cause.

Myocardial edema is not only one of the symptoms of pathological processes in the heart, but also independently causes deterioration in the course of the disease, increasing the risks of complications, often leading to irreversible changes in the tissue modeling [6], and increasing with arrhythmogenic potential [7,8], for example during acute myocarditis [9,10] and acute myocardial infarction [8,11,12]. The significant role of edema in cardiovascular diseases and acute cardiovascular events has only become distinct recently with the introduction of T2-weighted MRI images into widespread practice; nevertheless, the effect of edema on action potential and calcium wave propagation, as well as on proarrhythmic conductivity patterns, has never been studied.

The nature of arrhythmias in vivo is mainly determined by organic tissue damage associated with trauma, inflammation, and insufficient oxygen supply resulting in a local heterogeneity [13]. Furthermore, arrhythmias in the heart can be inherited, caused by cardiac channelopathies [14,15,16]. Stress may also be an additional factor that can aggravate the symptoms of arrhythmias originating from other etiologies [17]. Decades of studies in silico, in vitro, or ex vivo on perfused animal hearts have enabled the collection of a large amount of information regarding the potential mechanisms of arrhythmias and their association with various types of abnormal heart rhythm. Such mechanisms include re-entry, which resembles a spiral wave [18]. Electrical activity in the reentrant loop is characterized by the presence of a wave of impulse propagation to areas of the myocardium that have ceased to be excitable. This may occur due to a physiological block in the form of a scar, an area of necrotic cells, or a functional block caused an impaired cell excitation [19].

In addition to re-entry, numerous studies have investigated other patterns in excitable tissue, namely, wave meandering, wave break, spiral wave drift, and phase singularities [20,21,22,23]. The most generally accepted point of view is that re-entry potentially leads to the occurrence of tachyarrhythmias, while the exact mechanism of fibrillations, which are the most dangerous type of arrhythmias, still remains a controversial topic. Previously, spiral wave break was proposed to be the main cause of fibrillation-like arrhythmias, but a growing body of research has demonstrated [23,24] that wave breaks alone cannot trigger fibrillation; its initiation requires the presence of phase singularities (numerous excitation sites). Phenomenologically, when phase singularities are detected, wave breaks can also be observed, but the key factor is the multifocal excitations that maintain long-term fibrillation activity [19]. 

To simulate re-entry and fibrillation on perfused hearts or a cell monolayer, a functional blockade is caused by rapid point pacing or paired-pulse (S1–S2) protocol: the time delay between the pulses represents a vulnerable window, creating a spiral wave [25]. Pinning of a spiral wave can be induced by anatomic obstacles located in different parts of a monolayer [26]. In our study, different spatiotemporal patterns on a cell monolayer were obtained without specific pacing protocols, solely due to local heterogeneity, which corresponds to a more native modeling of organic tissue damage. Since edema can change the structure of tissue and lead to cell death, the resulting non-conductive area might be an obstacle for re-entry pinning or wave breaks.

Modeling myocardial edema in vitro is difficult due to a wide range of potential local changes that may occur in the tissue. The distribution of fluid between the intracellular and extracellular compartments, as well as between the vascular bed and the interstitial space, is determined by osmotic pressure, which, in turn, depends on the number of osmotically active particles in the solution. In biological fluids, osmolarity and osmolality are usually used to estimate osmotic pressure. Osmolarity is the concentration of particles dissolved in a liter of water, while osmolality is measured in a kilogram of water and is expressed in milliosmoles per kilogram (mOsm/kg). It is believed that osmolality provides a more accurate measure of the osmotic pressure of plasma, since blood plasma contains lipids and proteins, which normally occupy a certain volume and reduce the volume of the aqueous medium to 93% [27]. 

Sodium, its anions (bicarbonate and chloride), as well as glucose and urea make the greatest contribution to osmolality [28]. In the reviewed research papers, osmolality is regulated by changing the ionic composition (particularly, sodium) and further regulated with glucose, or by diluting the isosmotic solution with distilled water [29,30,31]. Reproducing physiological fluid and electrolyte imbalance is complicated by the fact that accurate measurement of such small local changes is impossible with existing methods; however, changes in the ionic composition of solutions may correspond to electrolyte imbalance in a real heart. 

Thus, the purpose of our study was to assess the influence of interstitial space osmolality on the calcium wave propagation dynamics using optical mapping of the cardiomyocytes in a monolayer. 

## 2. Materials and Methods

### 2.1. Ethical Aspects of Animal Experimentation

The experimental protocol was approved by the Local Ethics Committee of the Petrovsky National Research Center of Surgery (Approval No. 8, 23 September 2023). Humane welfare-oriented procedures were carried out in accordance with the Guide for the Care and Use of Laboratory Animals published by the United States National Institutes of Health (Publication # 85-23, revised 1996).

### 2.2. Neonatal Rat Ventricular Cardiomyocytes Isolation Protocol

Medium used during isolation: 

DMEM (Gibco, Thermo Fisher Scientific, Waltham, MA, USA) in the presence of 10% fetal bovine serum (STEMCELL, Cambridge, UK), 10 μg/mL streptomycin/penicillin (STEMCELL, Cambridge, UK) (DMEM10). 

DMEM in the presence of 5% fetal bovine serum, 10 µg/mL streptomycin/penicillin (DMEM5).

Leibowitz’s medium (BioinnLabs, Rostov-on-Don, Russia) in the presence of 10 μg/mL streptomycin/penicillin (L-15).

Isolation of primary neonatal cardiomyocytes from Sprague Dawley rats was carried out using a modified two-day Worthington protocol; the concentrations below were calculated for 10–15 rat pups. On the first day, the hearts of 0–3-day old rat pups were placed in a Hanks’ salts solution cooled to 4 °C. The hearts were then cleared of blood, atria, and aorta, and the remaining ventricles were placed in a 0.025% solution of trypsin (Invitrogen, Thermo Fisher Scientific, Waltham, MA, USA) in Hanks’ salts (Invitrogen, Thermo Fisher Scientific, USA) with the addition of 4.2 mM NaHCO_3_. The hearts were minced and left in the solution for 14–16 h at 4 °C. The next day, the heart pieces were placed in a 50 mL tube with 2 mL L-15 and 3 mL of 0.01% collagenase type 2 (STEMCELL, Cambridge, UK) dissolved in Hanks’ salts. The tube was placed in an incubator at 37 °C and 5% CO_2_ for 50 min, and every 10 min the tube was manually vortexed for 1 min. After 50 min, another 1 mL of collagenase solution was added, and incubation and vortexing continued for another 20 min. At the end of the incubation, the solution should become cloudy.

The suspension was then filtered through a 100 μm cell strainer (SPL Life Sciences, Pocheon-si, Republic of Korea) and centrifuged at 130 g for 5 min (LMC-4200R, Biosan, Riga, Latvia). The supernatant was removed, and 1 mL of L-15 was added to cells. Another 4 mL of DMEM10 was slowly added, drop by drop, into the tube. The resulting 5 mL of suspension with cells was seeded in a 25 cm^2^ flask for pre-plating for 1 h in an incubator at 37 °C and 5% CO_2_. An hour later, the suspension with unattached cardiomyocytes was transferred to a new tube. Live cells were counted using a Cell Counter (TC20, BioRad, Hercules, CA, USA) and trypan blue (BioRad, USA); the cells were seeded on 35 mm confocal dishes (SPL Life Sciences, Republic of Korea) pretreated with 20 μg/mL Fibronectin (H Fne-C, IMTEC, Moscow, Russia) solution, 1 million cells per dish. After 24 h, the cells were washed with PBS 3 times and 1.5–2 mL of DMEM10 was added again. After 48 h, the medium was replaced with DMEM5. After 3–4 days, the formation of a confluent monolayer and a contractile syncytium was verified using a light microscope.

### 2.3. Preparation of Solutions with Different Osmolality 

Tyrode’s isosmotic solution (1T) contained 140 mM NaCl (Serva, Germany), 5.4 mM KCl (Serva, Heidelberg, Germany), 1.8 mM CaCl_2_ (Serva, Germany), 1 mM MgCl_2_ (Serva, Germany), 10 mM Glucose (Serva, Germany), and 5 mM HEPES (Sigma-Aldrich, Burlington, MA, USA) in Milli-Q water. Hypoosmotic solutions (0.75T, 0.5T) were obtained by reducing the proportion of NaCl in the solution to 105 mM and 70 mM, respectively. The pH was adjusted to 7.4 using a 1 M KOH (Serva, Germany) solution.

### 2.4. Registration of Cell Area Changes 

Live cell imaging was conducted with Image ExFluorer (Live Cell Instrument, Namyangju-si, Gyeonggi-do, Republic of Korea) with an integrated environmental control system including CO_2_, O_2_, humidity control, and temperature. The prepared samples were placed in 1T solution in the incubator system at 37 °C and 5% CO_2_. Images were taken every 1 min for 90 min with a phase contrast plan semi-apochromatic correction 10x objective (Nikon Instruments, Tokyo, Japan) and 5.5 MP sCMOS camera (Nikon Instruments, Japan). After 5 min of incubation in 1T solution, the solution was changed to 0.75T or 0.5T. No external stimulation was used in order to stop the spontaneous contraction and register only changes in cell volume. Cell area was counted using ImageJ (v1.54g) at 0, 20, 40, 60, and 90 min (n = 13). The cell area was normalized to the area measured at 1T. The raw .nd2 files are available at: https://doi.org/10.5281/zenodo.13142633 (Published 31 July 2024).

### 2.5. Optical Mapping

A platinum point electrode and a reference circular electrode were used for the point stimulation of the cardiac tissue. Electrical stimulation parameters (PCGU-1000, Velleman, Gavere, Belgium) were pulse duration- 20 ms, -frequency- 0.8 Hz, 7 V. Calcium waves were recorded using Olympus MVX 10 (Olympus, Tokyo, Japan) fluorescence microscope equipped with an Andor iXon3 EMCCD camera (Andor Technologies, Belfast, UK) (recording speed 130 fps and resolution 256*256 pixels). Calcium wave imaging was performed on a monolayer of neonatal rat cardiomyocytes preliminarily stained with the calcium-binding dye Fluo-4 (F14201, Invitrogen, Thermo Fisher Scientific, USA) in a cultivation medium at a concentration of 1 µg/mL. The cells were incubated for 30–40 min without light at 37 °C and 5% CO_2_. After staining, the medium in the samples was changed to isosmotic Tyrode solution (1T). The solutions were changed according to the following scheme: the sample was initially placed in a 1T solution, and at the end of the measurement it was replaced with 0.75T and 0.5T, sequentially. After adding the solution with the lowest osmolality, the osmolality of the solution was successively raised from 0.5T to 1T. The measurement timepoints for each solution type were 0, 5, 20, 40, and 60 min. All experiments were performed at 37 °C. The schematic representation of the experiment protocol is shown in the Supplementary Materials.

### 2.6. Data Analysis

We processed 91 videos for 9 samples. The calcium wave propagation speed was calculated using ImageJ. Briefly, the background noise was first removed with Kalman filtering algorithm, and the filtered videos were smoothed with Gaussian blur. The contrast of the video was increased to the maximum and the required brightness was selected to obtain the clearest border. After preprocessing, a line was established transversely to a clear boundary of the wavefront, along which the speed was calculated. This algorithm was used previously [32].

Optical mapping of a cell monolayer provides highly reproducible results and allows the neglect of the curvature of the wavefront in three-dimensional space, reducing the number of factors that can affect the occurrence of various nonlinear patterns in excitable tissue. However, each sample has a different initial calcium wave speed, and these speeds are often not comparable to each other. Therefore, to compare experimental results, we normalized the speed to the average value during incubation at 1T for each sample. The data in all figures are expressed as mean (SD). The Friedman test was used to test whether there was a statistical difference in speed over time. For all results, *p* < 0.05 was considered significant. Visualization and statistical analysis were performed in Python (v3.10.13) using Matplotlib (v3.8.2), Seaborn (v0.13.2), and SciPy (v1.11.4).

In order to evaluate arrhythmogenicity and calcium dynamics spatial patterns, optical mapping data were phase processed with an open-source Python library, optimap (v0.2.2) [33,34]. Videos were normalized with sliding-window pixel-wise normalization (window size of 60 frames). Each wavefront was characterized by rotational activity (spiral waves), wave breaks, and phase singularities. Spiral waves were assessed with quantification of rotations and duration. The total number of spiral waves and phase singularity sites were calculated. A minimum of two rotations was used as a threshold to define a significant rotational activity, and three or more simultaneously activated sites were considered as fibrillatory activity.

## 3. Results

**Figure 1:**
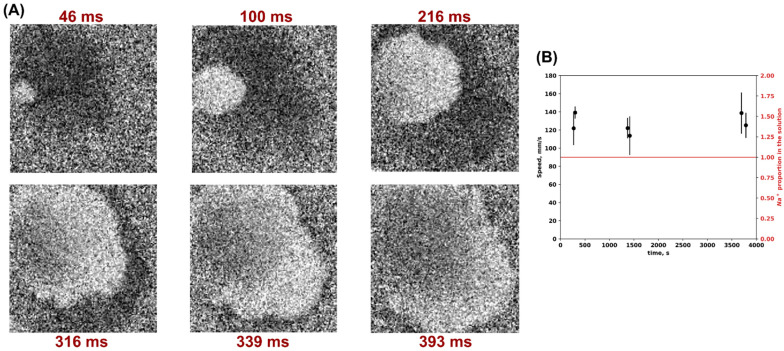
Calcium wave propagation dynamics at normal osmolality in 1T solution. (**A**) Optical mapping results, propagation of a single calcium wavefront over time. The dark area represents the zone of depolarization and calcium signal activation, and the light area represents repolarization. (**B**) Calcium wave propagation speed during long-term incubation in 1T solution. The mean (SD) values of calcium wave speed are indicated in black; the red line represents the Na+ proportion in the solution.

We tested the effects of calcium wave propagation in a monolayer of neonatal rat ventricular cardiomyocytes (NRVCMs) in 1T solution during long-term incubation (Figure 1). No changes in the calcium wave propagation patterns occurred in the samples in 1T solution, and we also did not find statistically significant differences in the calcium wave propagation speed during incubation for up to 1 h in Tyrode’s isosmotic solution.

**Figure 2:**
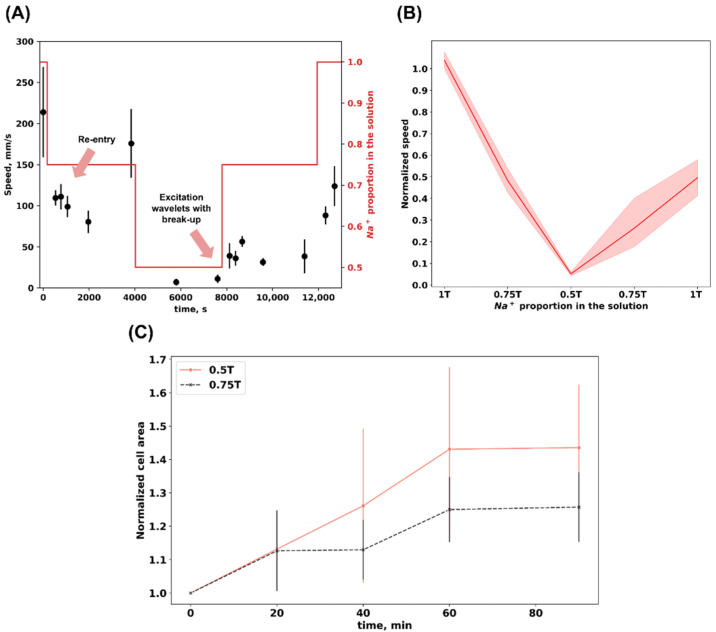
Video processing results of calcium wave optical mapping. (**A**) Calcium wave speed during successive changes of solutions 1T → 0.75T → 0.5T → 0.75T → 1T and the spatial patterns based on the results from one experiment without normalization. The mean (SD) values of calcium wave propagation speed are indicated in black; the red line represents the changes in Na^+^ proportion in the solution. Changes in dynamic patterns of wavefront are indicated with arrows. (**B**) Changes in normalized calcium wave propagation speed during successive changes of solutions were 1T → 0.75T → 0.5T → 0.75T → 1T for all experiments. The main trend line represents the mean value for each solution type, and the blurred area represents the 95% confidence interval. (**C**) Normalized cell area changes measured during 90 min in 0.5T and 0.75T solutions. The main trend line represents the mean value for each solution type and the error bar demonstrates standard error.

When the proportion of sodium ions decreased from 1T to 0.75T, the normalized speed decreased to 0.483 (0.078), and with a further lowering of the solution NaCl to 0.5T the speed amounted to 0.051 (0.012) (Figure 2B). It is important to note that, with a consistent increase in the osmolality of the solution, the calcium wave propagation speed did not restore to the initial values after an hour of incubation in solutions of 0.75T and 1T, rising to 0.262 (0.175) and 0.496 (0.117), respectively.

We also checked the dynamics of cell area in hypoosmotic solutions to check whether significant changes in cell size occurred in a monolayer (Figure 2C). In both solutions (0.75T and 0.5T) we observed cell size increase, and the measured area stabilized after 60 min of incubation in hypoosmotic solutions. The normalized area reached 1.25 (0.09) and 1.43 (0.17) for 0.75T and 0.5T solutions, respectively.

**Figure 3:**
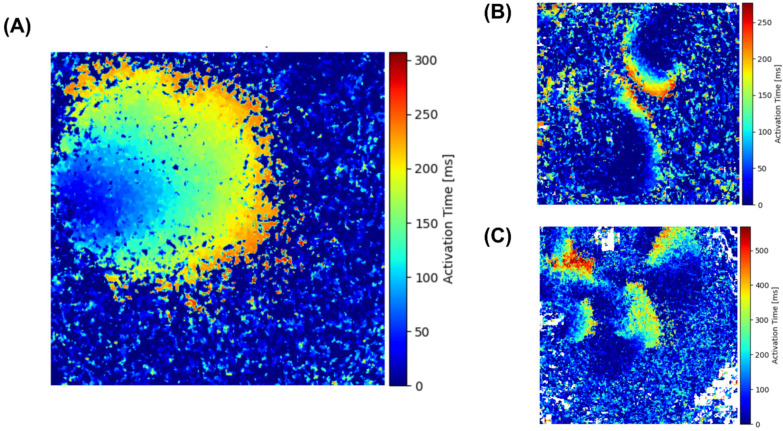
Activation maps for calcium waves in a monolayer of neonatal rat cardiomyocytes. (**A**) Activation map for 1T solution with normal wavefront. (**B**) Activation map for 0.75T solution and spiral wave formation. (**C**) Activation map for 0.5T solution and multiple wavelets of excitation formation.

The results from one of the optical mapping experiments are shown in Figure 2A, illustrating calcium wave propagation speed when changing the osmolality of solutions with a successive decrease from 1T to 0.5T and then an increase in the solution osmolality back to normal. In all samples, as previously mentioned, we observed a sharp decrease in the calcium wave speed when the NaCl concentration in the solution was reduced to 105 mM (0.75T). However, the dynamic patterns of wavefront between the solutions differed drastically. We indicated different nonlinear spatial patterns with arrows (Figure 2A). Spiral waves occurred in the first 4–5 min of incubation in a 0.75T hypoosmotic solution. In 0.5T solution (70 mM NaCl), the contractions almost completely disappeared, and after 1 h of incubation in the solution with a very low osmolality, multiple wavelets of excitation were obtained. The original videos with the discovered effects are presented in the Supplementary Materials. The activation maps of the three spatial patterns dependent on solution osmolality are demonstrated on Figure 3. Both spatial patterns were registered on other samples.

**Figure 4:**
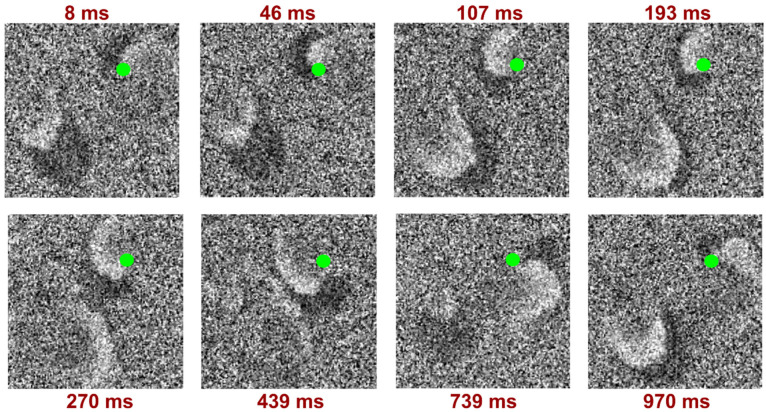
Calcium wave optical mapping in 0.75T solution. Spiral wave detected 755 s after the start of the experiment and 280 s after changing the solution from 1T to 0.75T. The dark area represents the zone of depolarization and calcium signal activation, and the light area represents repolarization. The green circle represents the point of spiral wave pinning.

Our results show that the restoration of normal contractile function of cardiomyocytes does not occur after normalization of osmotic pressure within 1 h. In order to assess the full recovery period, additional experiments are required. The detected spiral waves in the 0.75T solution appear in the first minutes of incubation with reduced osmolality and were recorded for the following 10 min; the time resolution and rotation of the spiral wave are demonstrated in Figure 4.

**Figure 5:**
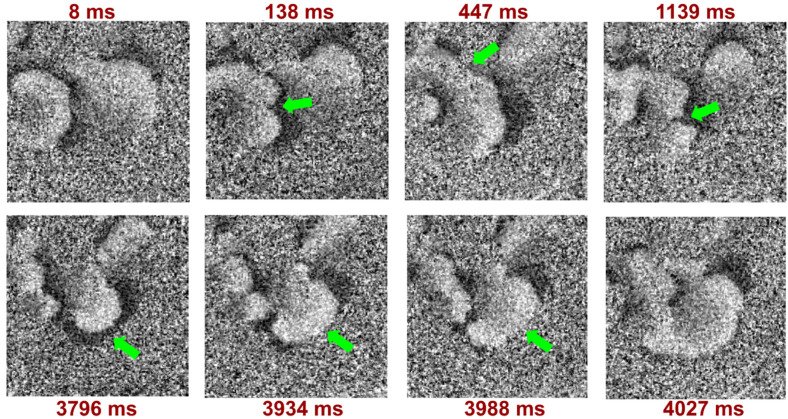
Calcium wave optical mapping results in 0.5T solution. Calcium multiple wavelets of excitation with wave breaks, detected 7603 s after the start of the experiment and 3583 s after changing the solution from 0.75T to 0.5T. The dark area represents the zone of depolarization and calcium signal activation, and the light area represents repolarization. The green arrows show areas with wavelet break-up.

Moreover, we registered the poorly studied multiple wavelets of excitation with wave breaks (Figure 5) which were not recorded previously on NRVCMs. The most stable and long-lasting calcium wave propagation pattern had four excitation sites, which appeared incrementally: from a short initial wave in the area with electrodes to gradually increasing chaotic excitation in several areas.

## 4. Discussion

In this study, cell edema was modeled in NRVCMs by reducing the osmolality of solutions. Previously, edema was analyzed only on isolated cardiomyocytes and the volume change was estimated in suspension. Certainly, the nature of the cell size changes in suspension, monolayer, and in a three-dimensional cell model can be very different given the pressure of surrounding cells. In our study, we estimated the change in the area of cells and found that the size of a cell reached its maximum after 60 min of incubation in a hypoosmotic solution. In a 0.75T solution, the change from the initial volume accounted for 1.25 (0.09) after 60 min, and in 0.5T reached 1.43 (0.17). The changes of cell size in a monolayer occur more slowly compared to cells in suspension according to the literature data [29], where 0.5T solution content was identical to our study. In our samples, we obtained a much larger deviation, which could also explain the presence of local heterogeneity leading to conductivity disturbance. Since we measured the area differences in hypoosmotic solutions, it is likely that changes along the z-axis may have occurred during the first minutes of incubation under such conditions.

The results of optical mapping showed a sharp decrease in the speed of calcium wave propagation (almost two times) 4–5 min after lowering osmolality to 0.75T. With a further decrease in osmolality to 0.5T, the contractile function almost completely stopped. This sensitivity of cardiomyocytes to changes in volume is in accordance with experimental data, including in vivo studies: with an increase in myocardium fluid content of just a few percent, cardiac output can decrease by 15 percent and more [35,36].

Calcium wave propagation patterns also differed between hypoosmotic solutions. We registered stable spiral waves after the first 5 min of incubation in 0.75T solution. Earlier studies based on mathematical models predicted the occurrence of various nonlinear dynamic patterns in excitable tissue [37,38], in particular, spiral waves in two-dimensional space (or scroll waves in three-dimensional space), their meandering, drift, and breakup. Recently, the existence of these spatial patterns was demonstrated in vitro and ex vivo [39,40,41,42,43] and associated with various cardiovascular diseases. Spiral waves, which potentially initiate tachyarrhythmias caused by re-entry [44], have been extensively studied from a biophysical perspective. Spiral wave formation requires several aspects: the presence of an area with blocked conduction in some direction, conduction of the impulse along an alternative path, delay in activation of tissue outside the block, and re-excitation of tissue proximal to the blocking area [37]. In addition, it is believed that re-entry is the initial stage, which can further develop into fatal ventricular fibrillation, including of post-myocardial infarction nature [45].

Spiral waves are initiated by a series of impulses that propagate during the refractory period of the previously excited area, thereby forming a functional blockade in a certain direction [46,47] or by creating a physical blockade in the form of some object or due to a site of non-excitatory cells, which results in spiral wave pinning [13,26]. Our results indicate that hypoosmolality also leads to stable spiral wave formation. The influence of hypoosmotic solutions on various calcium wave dynamics in the functional syncytium could be determined by cell edema, which was also shown in this study. We hypothesize that the rotating activity patterns may result from:

- Changes in the ion channels conductance, in particular, the activation of mechanosensitive channels.
- Changes in the mechanical properties of the sample:
  - a heterogeneous increase in cell volume with various swelling rates in different sample areas;
  - contractile apparatus disorder;
  - rupture of intercellular contacts.
- Changes in gap junction conductance disconnecting part of the culture from the functional syncytium.

To our knowledge, no studies have considered these factors in terms of edema-induced arrhythmias; several studies highlighted the arrhythmogenic potential of gap junction disruption. The authors showed that the treatment with gap junction inhibitors (1-heptanol (2 mM) or 18-b-glycyrrhetinic acid (30 mM)) led to changes in beating cooperativity and the propagation pattern in the cardiomyocyte monolayer [48]. Another research study demonstrated that local heterogeneity in the Cx43 distribution correlated accurately with conduction block and changes in maximal upstroke rate of rise of the action potential [49]. The data indicate that the cellular structure, remodeling of the extracellular matrix, and gap junction distribution may represent potential sites for a block in the transverse direction, which is especially relevant in chronic myocardial infarction or ventricular hypertrophy. Of course, the question of exactly which factors or their combination can influence the proarrhythmogenic potential of edema requires further study, and the literature emphasizes the significant role of myocardial edema in the remodeling of the local tissue structure.

In addition to spiral waves, multiple wavelets of excitation were registered after 60 min of incubation in 0.5T solution, which corresponds to the maximum increase in cell size. We believe that such a pattern might represent fibrillatory activity. The nature of fibrillation still remains controversial; previously it was believed that the fibrillation-like state arises due to the appearance of the spiral wave itself [50]; then, it was proposed that fibrillation was caused by other nonlinear spatial patterns, in particular, the spiral wave breakup [51,52,53,54,55].

The studies devoted to ventricular fibrillation have demonstrated the appearance of multiple wavelets of excitation, which may not have rotating activity, but they are characterized by phase singularities, regions in which the activation state cannot be determined, surrounded by a continuum of activation states ranging from fully activated to fully recovered. Moreover, despite the fact that wave breakups are phenomenologically similar to phase singularities, both phenomena represent different patterns [20]. An in silico approach showed spiral wave breakup [22,54,56,57].

Fibrillations in vitro and ex vivo are usually caused by a series of impulses which stimulate heterogeneous cardiac tissue with a period shorter than the refractory period [58]. Experimentally, several spatial patterns were previously registered: calcium wave break collision, which did not transform into multiple wavelets of excitation state [25], phase singularity dynamics of membrane potential on perfused rabbit hearts [59], multi-wave reentrant patterns of membrane potential on NRVCMs [47], and vortex filaments of membrane potential on perfused rabbit and pig hearts [60].

As previously discussed, local heterogeneities are omnipresent in the myocardium, but it is rarely discussed that cardiomyocytes in the tissue are separated by extracellular space filled with interstitial fluids [61] and changes in osmotic pressure can significantly affect myocardial remodeling. At the same time, gap junctions can influence the fibrillation pattern that occurs in the heart: in rat hearts, gap junction coupling was shown to support rotating activity, while, with increasing degrees of uncoupling, the propagation of the membrane potential became more stochastic, leading to the formation of multiple wavelets with no rotating activity [62,63]. In our study, the successive decrease in osmolality also showed a transition between spiral waves and multiple wavelets of excitation.

Thus, our study showed that, in a cellular model, hypoosmolality can potentially lead to fatal ventricular arrhythmias and might be associated with myocardial edema: at 0.75T spiral waves can appear, whereas multiple wavelets of excitation with wave breaks occur in solutions with a very low osmolality (0.5T), potentially being the main cause of fibrillations. It is likely that the susceptibility to arrhythmias in post-infarct tissue is associated not only with the presence of scars, but also with the area of swollen cells, which is larger in the acute phase of myocardial infarction and may be characterized by additional local heterogeneity.

There are a number of limitations in our study that require further research. The purpose of our study was to register nonlinear spatial patterns in edematous NRVCMs and identify their connection with dangerous ventricular arrhythmias, as well as collect statistical data on the calcium wave propagation speed under various solution osmolality, which can later be used for mathematical simulations. In general, an in silico approach may allow the preliminary testing of hypotheses regarding the biological factors that lead to changes in conduction patterns during cell swelling. However, additional experiments need to be carried out for more accurate understanding of the mechanisms behind the observed phenomena.

In our work, we modeled edema by changing the Na^+^ content in a modified Tyrode solution. The osmotic pressure of the original Tyrode solution and its ionic composition are close to the blood plasma and interstitial space. Our approach was determined by several factors. Firstly, Na^+^ is the dominant extracellular electrolyte and greatly determines serum and interstitial osmolality and, consequently, fluid volume in different compartments [64]. Secondly, low plasma osmolality and hyponatremia occur in cardiovascular diseases, are common electrolyte disorders in hospitalized patients with acute decompensated heart failure, are independently predictive of worse post-discharge mortality [64,65,66], and require constant monitoring during the perioperative period [67].

Moreover, cardiovascular diseases are caused by disturbances in the hydrostatic pressure in blood vessels and are characterized by microvascular obstruction and ischemia [68]. Acute myocardial injury is followed by water dispersion in the interstitial and intracellular interstitial spaces, resulting from vasodilatation and increased capillary permeability [69]; excessive fluid flow from plasma may decrease sodium content in the interstitium as well. Hemodynamic changes in the heart also result in ventricular tachycardia or ventricular fibrillation [70,71,72,73].

Earlier work that studied the effect of osmotic stress on sarcolemmal disruption showed that the observed effects were not associated with low Na^+^ concentration present in the hypoosmotic buffer [30]. In our study, we also observed that the calcium wave propagation speed does not recover to its initial values even after an hour after incubation in a 1T solution. We assume that it is most likely that the effect of edema on cells is more damaging than a change in the concentration of Na^+^, since the characteristic times for the cells to respond to ionic changes are much shorter than for the restoration of impaired intercellular interaction. For this reason, we believe that the effects we found could be attributed primarily to edema; however, in order to completely exclude the effect of low sodium content on calcium wave propagation properties, it is necessary to conduct additional experiments with low sodium solutions, where osmolality is compensated by organic solutes.

## 5. Conclusions

This work provided several important results regarding the arrhythmogenic potential of myocardial edema. We demonstrated a sharp decrease in the calcium wave propagation speed with a decrease in the solution osmolality due to the NaCl content; with a decrease in the proportion of sodium from 140 mM to 105 mM, the normalized speed decreased by more than half to 0.483 (0.078), and with 70 mM, NaCl decreased to 0.051 (0.012). In the solution with the lowest osmolality, contractions almost stopped, which demonstrates the high sensitivity of cardiomyocytes to volume changes.

In addition, for each solution we obtained different nonlinear spatial patterns that correspond to several types of arrhythmias. Spiral waves were detected after 4–5 min of incubation in the solution with 105 mM NaCl, and after an hour of incubation in a solution with 70 mM NaCl, multiple wavelets of excitation appeared, which were recorded in a two-dimensional monolayer under hypoosmotic conditions that could be associated with cell edema. We hypothesize that the observed effects may be associated primarily with changes in gap junction conductance, disruption of intercellular contacts, and cytoskeletal disorders; however, additional research is necessary to explore the process mechanistically.

## Data Availability

The original data that support the findings of this study are included in the article and supplementary material, further inquiries can be directed to the corresponding author, D. G. Kiseleva.

## Supplementary Materials

The following supporting information can be downloaded at: https://www.mdpi.com/article/10.3390/biomedicines12081770/s1, Video S1: Normal wavefront 1T; Video S2: Spiral wave at 0.75T; Video S3: Multiple excitation wavelets at 0.5T; Figure S1: Schematic representation of the experiment protocol.
