# Supplementary figures and images for: Arrhythmogenic Potential of Myocardial Edema: The Interstitial Osmolality Induces Spiral Waves and Multiple Excitation Wavelets
Source: Biomedicines. 2024 Aug 6;12(8):1770. doi: 10.3390/biomedicines12081770 (PMC11351629; doi:10.3390/biomedicines12081770)

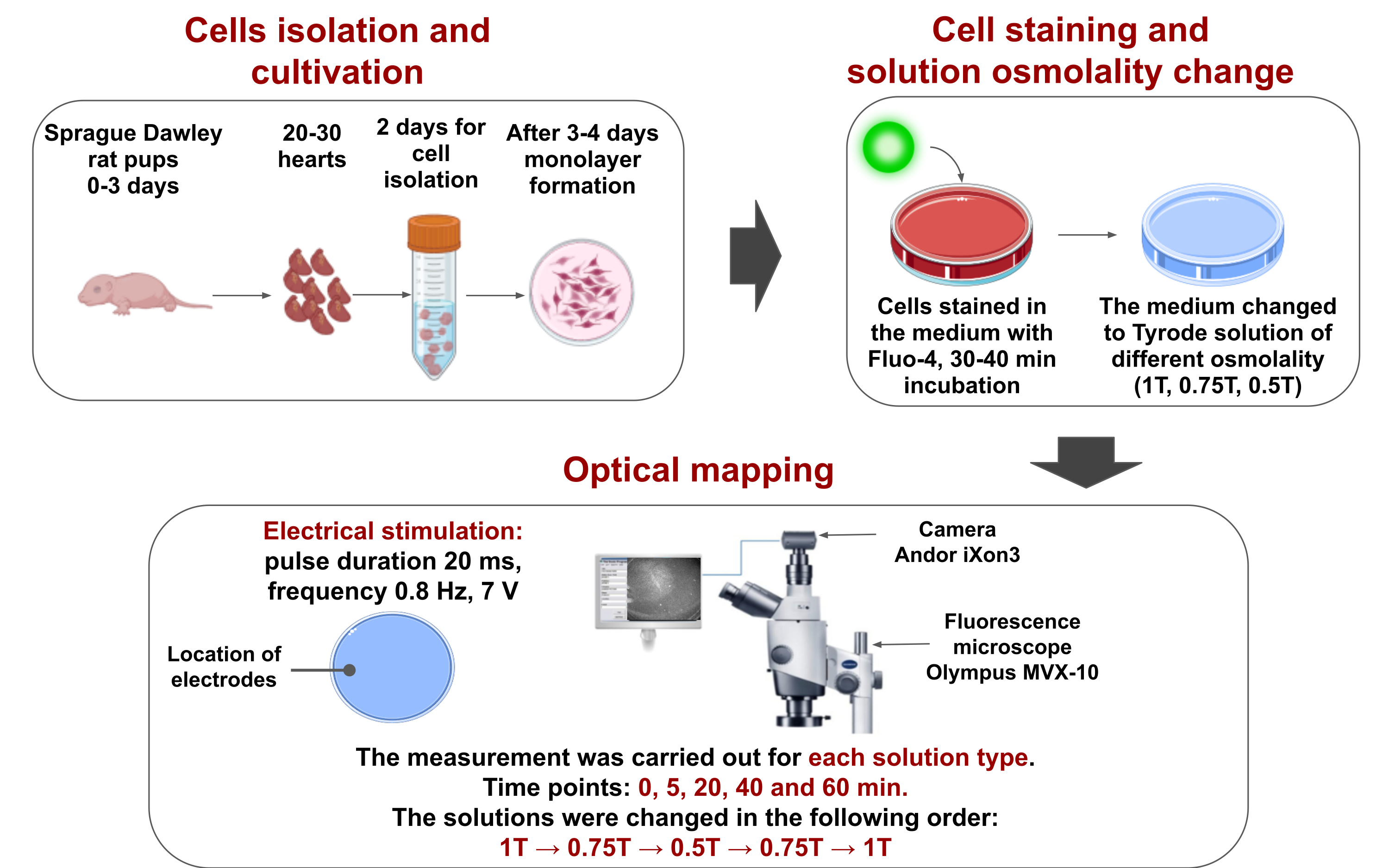

Supplement: Supplementary file 1 [file biomedicines-12-01770-s001.zip › Figure S1.tiff]
